# The Impact of SMS-Based Interventions on VMMC Uptake in Lusaka Province, Zambia: A Randomized Controlled Trial

**DOI:** 10.1097/QAI.0000000000001040

**Published:** 2016-10-06

**Authors:** Kevin Leiby, Alison Connor, Landry Tsague, Crispin Sapele, Albert Kaonga, Joshua Kakaire, Paul Wang

**Affiliations:** * IDinsight, Lusaka, Zambia;; † UNICEF , Regional Office, West and Central Africa Region, Dakar, Senegal;; ‡ CHAMP, Lusaka, Zambia;; § Zambia Ministry of Community Development, Mother and Child Health, Lusaka, Zambia and UNICEF Zambia Office; M&E Unit, Lusaka, Zambia.

**Keywords:** male circumcision, HIV, mHealth, SMS, counseling, behavior change

## Abstract

Zambia has high HIV prevalence and low voluntary medical male circumcision (VMMC) rates, heightening the need for effective VMMC demand generation strategies for HIV prevention.

**Methods:**

A 3-arm randomized controlled trial measured the impact of 2 short message service (SMS) campaigns on self-reported and verified VMMC uptake over 6 months in Lusaka Province. The study enrolled 2312 uncircumcised males aged 15–30 previously subscribed on Zambia U-Report, an existing SMS platform providing confidential, free counseling services relevant to HIV and other sexually transmitted infections. Participants in the “Conventional” campaign group received a standard package of messages promoting VMMC. Messages sent to the “Tailored” campaign group were targeted at participants' intention level to get circumcised. The control group had routine counselor access through SMS. Data were collected using SMS surveys, and verification of self-reported VMMC uptake used health facility client data.

**Results:**

Six-month self-reported VMMC uptake was 11.6%, 12.6%, and 10.4% in the Conventional, Tailored, and control arms, respectively; verified uptake was 1.8%, 1.1%, and 1.5%. Using multivariate logistic regression, the adjusted odds ratio of self-reported VMMC uptake was 1.17 (95% CI: 0.80 to 1.72) in the Conventional campaign arm compared with the control arm and 1.24 (95% CI: 0.84 to 1.81) in the Tailored campaign arm. The adjusted odds ratios of verified VMMC uptake in the Conventional and Tailored campaign arms were 1.34 (95% CI: 0.45 to 4.02) and 0.67 (95% CI: 0.20 to 2.23), respectively.

**Conclusions:**

Neither SMS campaign had statistically significant impact on VMMC uptake compared with routine SMS counseling. Future research is necessary to fully understand the potential of SMS-based tools for VMMC demand creation.

## INTRODUCTION

In 2014, there were 2 million new HIV infections globally, bringing the number of people living with HIV to 36.9 million.^1^ Voluntary medical male circumcision (VMMC) has been shown to reduce the risk of female-to-male HIV transmission by around 60%.^2–5^ As a result, the World Health Organization and the Joint United Nations Programme on HIV/AIDS recommend including VMMC as a component of a comprehensive HIV prevention package in 14 priority sub-Saharan African countries with high HIV and low VMMC prevalences.^6^

As a priority country, Zambia has rapidly expanded efforts to deliver safe VMMC services. With service capacity exceeding demand, policymakers have placed greater emphasis on identifying evidence-based strategies for VMMC demand generation.

Mobile health interventions and short message service (SMS) communication offer unique opportunities to reach large groups of people quickly and inexpensively. However, results of studies looking at the effectiveness of these interventions as behavior change tools are mixed. One review of 9 randomized and quasi-experimental studies of SMS interventions to influence disease prevention and management found 8 to be effective.^7^ Additional studies have either found or suggested impact of SMS-based interventions on health knowledge acquisition,^8,9^ adherence to appointments,^10,11^ adoption of safer health behaviors,^12^ and uptake of and adherence to HIV biomedical interventions such as antiretroviral therapy regimens, condom use, and HIV testing.^13–16^

Other studies, however, have produced less promising results. Jamison et al^17^ found that an information service in Uganda providing automated responses to SMS questions about sexual health had no impact on knowledge or attitudes, and increased rather than decreased unsafe sexual behavior among men. Odeny et al^18^ examined the effect of a series of text messages on deterring the resumption of sexual activity among recently circumcised men but found no effect compared with a control group. A systematic review found limited evidence that mobile phone messaging on preventive health care had any effect on improved health status or behavior outcomes.^19^

This study measured the effects of 2 SMS campaign strategies over a 6-month period using an existing SMS platform, Zambia U-Report, on VMMC uptake compared with a control group with routine platform access.

## METHODS

### Study Setting

This study took place in urban Lusaka and periurban Chongwe districts in Lusaka Province, Zambia from May to October 2014. Lusaka Province was selected because of both high phone ownership among young people and high HIV infection rates. HIV prevalence among Zambian males aged 15–49 in Lusaka Province is 19%.^20^ Neighboring Chongwe district was included at the request of policymakers interested in potential for impact in periurban and rural locations.

The Government of Zambia and cooperating partners provide free VMMC services at many public health facilities. Throughout the 6-month study period, VMMC service was provided regularly at over 20 sites in Lusaka and 1 central site in Chongwe, with expanded service at additional facilities and mobile sites during national VMMC campaign periods in early May and August.

### U-Report Platform

This study took place among subscribers of the existing “Zambia U-Report” national SMS platform (http://www.zambiaureport.org), which provides free, confidential, and interactive counseling to adolescents and youths with trained 24-hour counselors on HIV/AIDS and other sexual and reproductive health topics. Program managers can also send mass polls or informational messages to subscribers. At the end of 2014, 75,000 subscribers had self-enrolled onto the platform. Subscriber phone numbers are not accessible to the counselors or program managers, making the platform strictly confidential.

### Intervention

This study tested the impact of 2 different campaign strategies that affected the mix of messages sent, the “Conventional” and “Tailored” campaigns. Each campaign sent packages of 7 SMS messages every 2 months over a 6-month period (21 messages) between May and October 2014. Messages were developed that aligned with Zambia's National VMMC Communication and Advocacy Strategy^21^ and were informed by previous U-Report polling on VMMC uptake barriers. They provided information and prompted participants to learn more, engage counselors, and go for VMMC.

**FIGURE 1.**
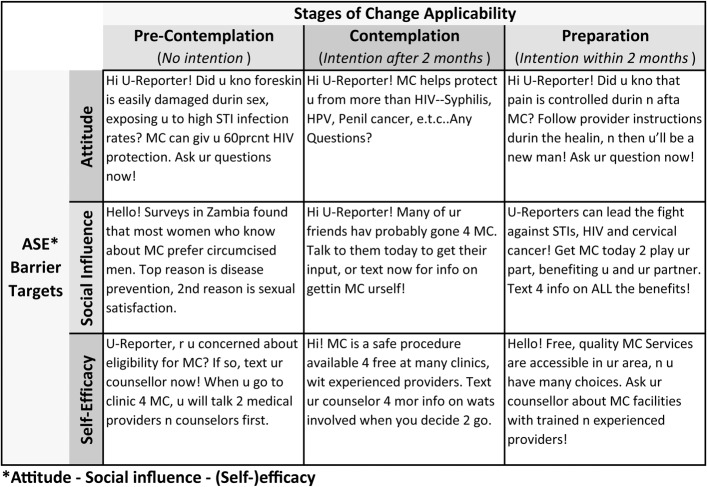
Example messages.

Messages were categorized along 2 dimensions of the theory of change (Fig. 1). The first dimension delineated messages by relevance based on different self-reported intention levels—precontemplation (no intention), contemplation (intention beyond 2 months), and preparation (intention within 2 months) according to the stages of change framework.^22^ The second dimension delineated messages by their behavior change tactic according to the attitude–social influence–self-efficacy framework.^23^

The “Conventional” campaign consisted of the same mix of messages across both dimensions. The “Tailored” campaign consisted of messages addressing each attitude–social influence–self-efficacy barrier but targeted to participants' specific self-reported stage of change. Participants with lower intention mainly received simpler VMMC information; participants reporting greater intention mainly received information related to accessing VMMC services and undergoing the procedure.

### Study Design and Sample Size

The study was a 3-arm randomized controlled trial (RCT), testing the impact of the Conventional and Tailored SMS campaigns on VMMC uptake compared with a control arm. Control participants did not receive campaign messages relating to VMMC but had routine access to the U-Report platform and could still engage counselors on any topic, including VMMC.

All U-Report subscribers who self-registered on the platform as male between 15 and 30 years in Lusaka or Chongwe districts (11,447 total) received a baseline SMS survey. Those who responded to the baseline survey that they were uncircumcised (2312) were enrolled in the study. The study sample was stratified by district (Lusaka or Chongwe), age (<18 or ≥18), and self-reported VMMC intention (within 2 months or not). Participants were then randomly assigned to one of the 3 study arms.

The evaluation's *a priori* sample size calculation before accounting for covariates estimated that a 2.5% effect size could be detected (beta = 0.20; alpha = 0.05) in either treatment arm assuming 1% VMMC uptake in the control arm and a sample size of 2040 after accounting for 20% attrition. However, because fewer subscribers met the eligibility criteria, the final sample for analysis was 1652 participants (after loss to follow-up and revealed ineligibility). Back-power calculations indicated that with this sample size, we could detect a 2.8% difference without accounting for covariates. Calculations were performed using Optimal Design version 3.0 (Optimal Design Software, Arlington Heights, IL) and Stata 12 (StataCorp LP, College Station, TX).

### Definition and Measurement of Outcomes

There were 2 primary outcomes: self-reported VMMC uptake and verified VMMC uptake because we assumed that self-reported data would overestimate true uptake, whereas verified data would underestimate it because of limited data for verification. In addition, verification of uptake could only occur for participants reporting uptake at a facility in the study area.

A participant was considered to have experienced a self-reported outcome if he reported uptake that was not contradicted in subsequent follow-up surveys. Uptake was “verified” if a participant's U-Report registration and self-reported uptake data matched with client data from health facilities. Because the U-Report platform is confidential, the study team only had access to the last 5 digits of participants' registered phone numbers, their age, and their neighborhood. The criteria used required that the site of uptake matched, as well as at least 4 of the 5 phone digits, with a low probability of that match occurring by chance (thresholds used were: <1% for 5 digits; <0.5% for 4 digits) as estimated using neighborhood and age criteria.

A secondary outcome was engagement with counselors, defined as participants sending questions or messages to U-Report outside data collection survey responses.

### Data Collection

The main method for collecting information from participants was SMS surveys. A baseline survey was used to screen for eligibility and follow-up surveys were sent to enrolled participants at 2, 4, and 6 months after baseline. Each survey was similarly structured around a question about circumcision status. Participants who responded that they were circumcised were asked where and when they had the procedure; participants who responded that they were uncircumcised received questions on intention to go for VMMC. Surveys also included questions about phone usage, tribe, education, relationship status, and circumcised family members. Every participant who completed a survey received an airtime incentive worth 2 Zambian kwacha (ZMW; ∼0.32 USD)—enough to cover about 6 SMS messages or about 3 minutes of talktime.

Client data from public and private health clinics providing VMMC in the study area were used to verify self-reported uptake during the study period. A field team collected limited data (procedure date, partial phone numbers, age, and neighborhood) from client intake forms. These data were entered into an electronic database using EpiData Entry v3.1 (The EpiData Association, Odense, Denmark).

Finally, participant registration information (sex, age, and area of residence) and messaging data between the anonymous participants and counselors were collected from the U-Report platform.

### Ethical Considerations

The ERES Converge IRB in Zambia approved the evaluation's research protocol, and Zambia's Ministry of Health and Ministry of Community Development, Mother, and Child Health granted authorization to conduct the research.

Enrollment on the U-Report platform is voluntary and noncoercive. Subscribers are typically made aware of U-Report through mass media and their peers. All participants were enrolled on the platform at the start of the study. Because study participants were anonymous and SMS campaigns and polls are regular features of the U-Report platform, no additional consent was obtained from study participants. Participants could decline to complete any of the SMS surveys and still received the airtime incentive. In addition, participants could unsubscribe from the U-Report platform at any time, an option available to all U-Report subscribers.

To verify circumcision of anonymous U-Report subscribers, we collected limited deidentified data from client records at all health facilities in the study area. Because all data were collected without identifiers and only a limited number of study staff accessed records under the supervision of facility staff, informed consent was not sought from VMMC clients. These procedures were approved by the ERES Converge IRB, Zambia's Ministry of Health, Ministry of Community Development, Mother, and Child Health, all partners providing VMMC services at study facilities, and the study sponsor.

### Statistical Methods

A logistic regression model was used to estimate the odds of both self-reported and verified VMMC uptake among participants in each of the campaign arms compared with the odds among those in the control arm. In addition to bivariate models, odds ratio estimates were adjusted for key covariates collected from registration data: age (under 18 at baseline) and district; from SMS surveys: baseline intention of VMMC within 2 months, circumcised family members, affiliation with tribes reporting high uptake during the campaign, and the number of follow-up survey responses; and from client data: estimated “verifiability” of self-reported data. Statistical significance was assessed using an alpha of 0.05. Finally, linear regression was used to estimate the relationship between counselor engagement and VMMC uptake.

All analyses were completed using Stata 12.

## RESULTS

**FIGURE 2.**
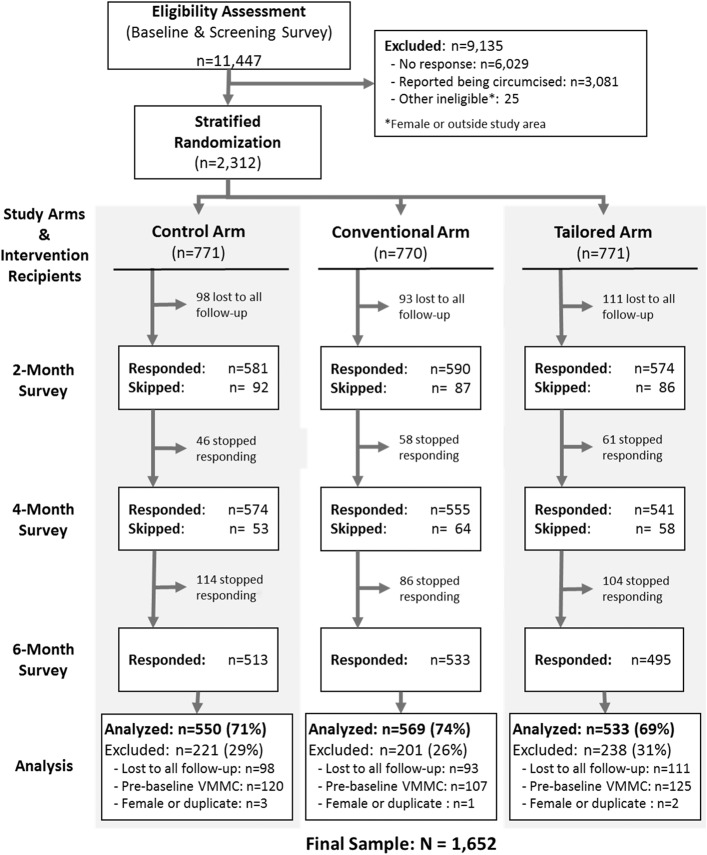
Sample survival.

There were 2312 U-Report subscribers enrolled in the evaluation. Throughout the study, some participants revealed or strongly suggested in survey responses and messages to U-Report counselors that they were actually ineligible because they were already circumcised at the campaign launch date (n = 352), female (n = 6), or enrolled in the study on multiple phone numbers (n = 4). This revealed ineligibility was not statistically significantly associated with study arm. These 362 (15.7%) participants were excluded from the analysis. In addition, 302 participants (13.1%) did not respond to any of the follow-up surveys; those responding to at least 1 survey were included. The final sample available for analysis was 1652 (71.5% of the original sample) (Fig. 2).

Participant characteristics were similar across study arms (Table 1). Participants who failed to respond to surveys or were otherwise dropped from the analysis were similar to respondents and also across arms based on limited data available (data not shown).

**TABLE 1.**
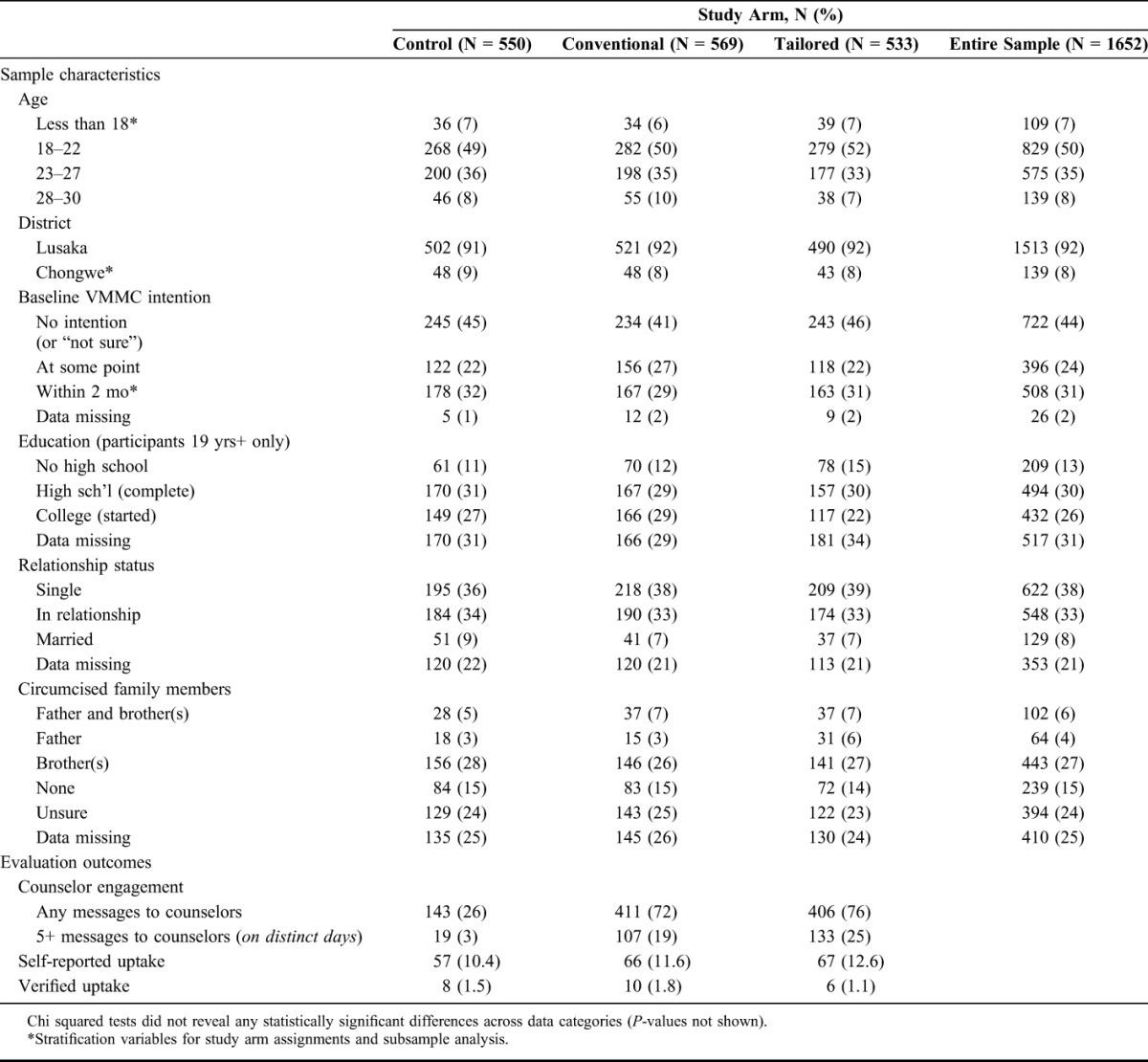
Sample Characteristics and Evaluation Outcomes

Most of the participants lived in Lusaka and were between 18 and 27 years. In addition, 39% of survey respondents over the age of 18 had started or completed postsecondary education. Slightly more participants were in relationships or married than were single. A high percentage of survey respondents (48%) reported immediate family members who were circumcised. Over half (55%) reported baseline intention to go for VMMC at some point, with 31% reporting intention to go for VMMC within 2 months.

### Self-Reported Outcomes

A total of 441 participants reported that they were circumcised at some point during the 6-month follow-up. However, 190 of those reports (43%) satisfied the outcome criteria in that they were not contradicted in subsequent reports. Self-reported uptake by the end of the 6-month follow-up period was 11.6% in the Conventional arm and 12.6% in the Tailored arm, compared with 10.4% in the control arm (Table 1).

Conventional arm participants had an adjusted odds ratio of 1.17 (95% CI: 0.80 to 1.72; *P*-value = 0.41) compared with those in the control arm; Tailored arm participants had an adjusted odds ratio of 1.24 (95% CI: 0.84 to 1.81; *P*-value = 0.28) (Table 2).

**TABLE 2.**
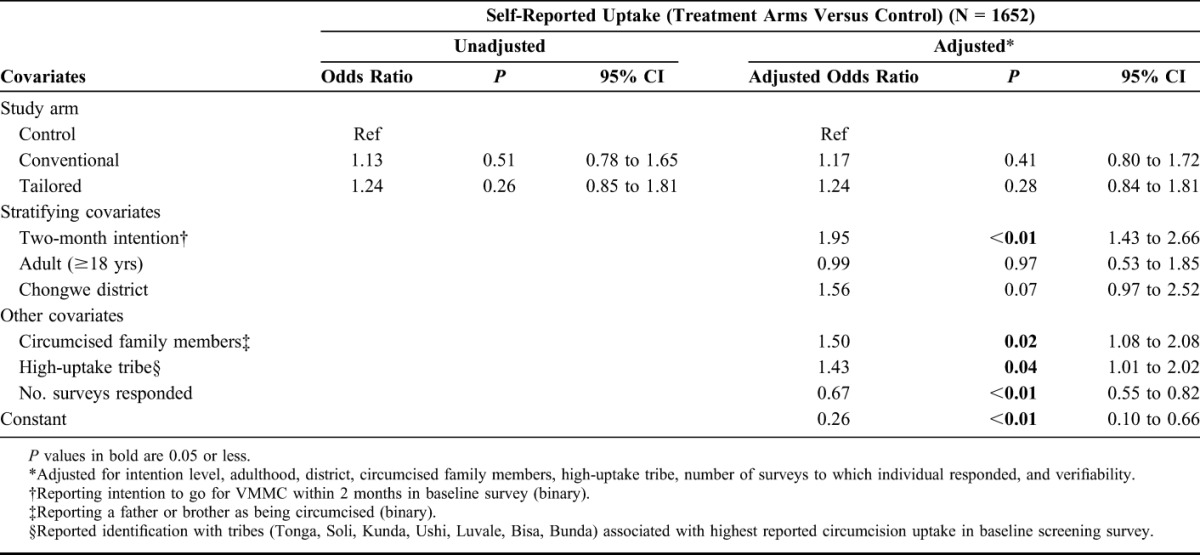
Regression Results for Self-Report Outcome

### Verified Outcomes

Only 24 (12.6%) self-reported outcomes were verified using client data, providing a small sample with which to confidently assess the impact of the 2 interventions. Overall, 1.5% of control arm participants were verified going for VMMC during the study period versus 1.8% in the Conventional arm and 1.1% in the Tailored arm (Table 1). Neither intervention arm had a statistically significant adjusted odds ratio nor were the results indicative of impact by either arm (Table 3).

**TABLE 3.**
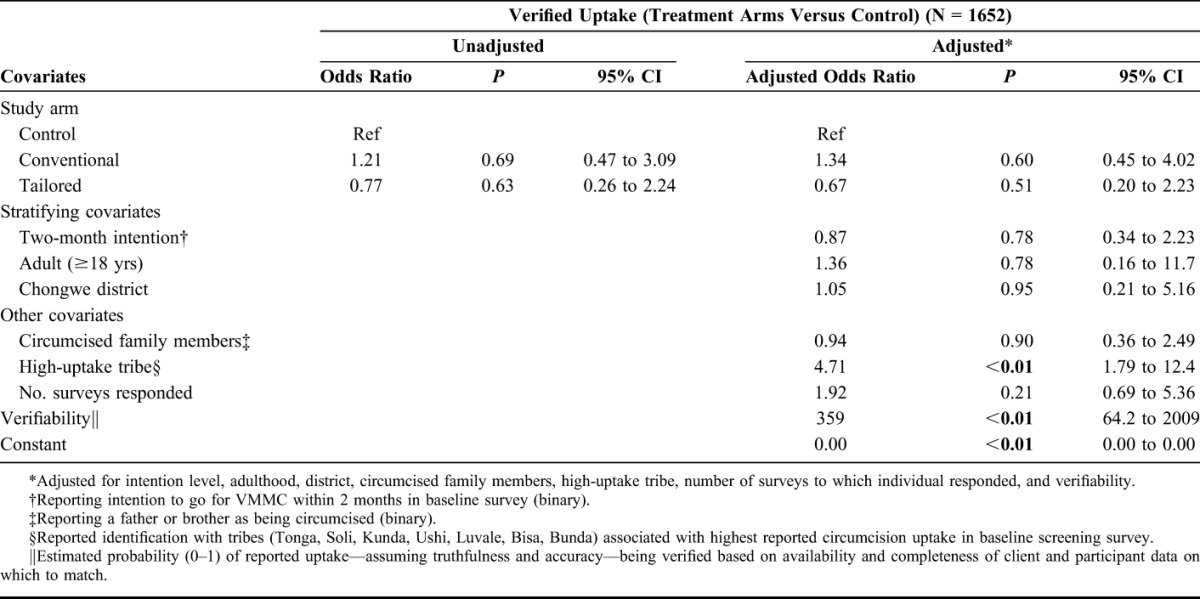
Regression Results for Verified Outcome

### Counselor Engagement

The SMS campaigns yielded a high proportion of treatment-arm participants messaging counselors during the campaign—largely but not exclusively about VMMC—revealing high demand for information or counselor support (Table 1). Outside SMS survey windows, 74% of treatment-arm participants messaged a counselor at least once versus 26% in the control arm. In the treatment arms, 22% of participants messaged counselors on at least 5 occasions compared with 3% in the control arm. These differences were statistically significant (*P*-value < 0.01) using a linear regression. Notably, neither binary nor discrete (number of messages) measures of SMS engagement with counselors were associated with VMMC uptake outcomes in the treatment arms (*P*-value = 0.83) using a linear regression.

## DISCUSSION

This study used a RCT to test the impact of 2 SMS campaign strategies on VMMC uptake compared with routine access to an existing health counseling platform. Not only did it test the impact of a standard set of messages, but it also attempted to target messages to participants' self-reported intention levels. The findings of this study indicate that neither the Conventional nor the Tailored SMS campaign strategy increased demand for VMMC over a 6-month period. Although the campaigns stimulated engagement with SMS counselors, this did not seem to translate into greater uptake.

The campaigns aimed to positively influence health-seeking behavior by providing information and encouraging uptake of VMMC. However, material barriers and opportunity costs, such as time away from work and from sexual activity, could not be addressed by this intervention and may have been more influential drivers of behavior change than lack of information or encouragement. Other contexts in which SMS communication has proven impactful, such as smoking cessation or adherence to antiretroviral regimes,^12,24^ require sustained support over time, whereas going for VMMC requires a bold one-time decision. Qualitative research in Zambia and Zimbabwe that mapped the pathways to circumcision uptake suggests that short-term consequences of VMMC such as opportunity costs or implications for sex life stall or avert uptake, especially for men in their twenties who are working or in relationships.^25^ These late-stage barriers could have been most relevant to the study population if platform subscribers were already largely aware of HIV prevention and concerned about sexual health.

Another major finding is that self-reported uptake of VMMC was an unreliable outcome, even after controlling for inconsistent reporting. An unrealistic proportion of participants (11.5%) reported circumcision uptake without any contradiction across multiple surveys, and an additional 15.2% reported contradictory information. Another study in Zambia similarly found that in-person reports of circumcision status were unreliable, and the SMS medium of data collection may have magnified that unreliability.^26^ Social desirability bias may have played an important role in over-reporting, and it is also possible that participants who made up their mind to obtain the procedure (but had not yet done so) were more likely to report positively. Either way, research looking at VMMC uptake should attempt to verify it to the extent possible.

To the best of our knowledge, this was the first RCT of an SMS-based strategy to generate demand for VMMC. In addition to the rigorous study design, a strength of the study was its use of a verified outcome. In addition, despite not achieving the desired sample size, the final sample was large enough to detect policy-relevant levels of impact of about 2%–3% greater uptake.

The study was not without its limitations. First, the verification methodology was constrained by participant anonymity, limited data, and gaps in health facility records despite frequent data quality monitoring and reinforcement. In addition, incomplete or falsely reported aspects of uptake (eg, site and date) could make verification unlikely or impossible. Treatment-arm participants received information on VMMC and could have reported their status more accurately. Although we attempted to drop participants who later revealed ineligibility, some ineligibility may not have been detected, and others may have been mistakenly dropped. These factors should not have been biased across arms, but they introduced additional uncertainty into the data.

The SMS surveys themselves increased counselor engagement in all study arms, including the control. Furthermore, the control group still had access to U-Report counselors. It is possible that the counseling function—with prompting from surveys—was sufficient for influencing behavior, but the study was only designed to measure the marginal and interactive effect of campaign messaging.

Next, 13% of the enrolled sample was lost to follow-up. Moreover, many participants (40%) failed to respond to all study surveys, so data over the entire 6-month study period was partial. Although overall response rates were better than expected, missing data meant that the study window in which to measure uptake was reduced for some participants, for example, who did not respond to the study's 6-month survey.

Finally, external validity was affected by the self-selected sample of Zambia U-Report subscribers who are likely wealthier, more educated, and more concerned or aware of sexual health issues than the general population. The interventions evaluated did not occur in isolation of significant VMMC promotion through mass media and community mobilization activities 3 years into the government's national VMMC program.

## CONCLUSIONS

This evaluation measured potential impact of 2 SMS-based campaign strategies on VMMC uptake. Neither campaign strategy was effective at generating demand that translated into uptake after 6 months. The campaigns were, however, effective at increasing engagement with SMS counselors on the U-Report platform, enabling participants to access answers to their specific questions about VMMC or to receive individualized support. Thus, although SMS-based campaigns on U-Report are unlikely to be effective as stand-alone, marginal interventions for VMMC demand creation, they may still play a role in decision-making processes. Further research may examine the potential of SMS interventions alongside other approaches.
